# Supplementary material for: Targeting ABCC6 in Mesenchymal Stem Cells: Impairment of Mature Adipocyte Lipid Homeostasis
Source: Int J Mol Sci. 2022 Aug 16;23(16):9218. doi: 10.3390/ijms23169218 (PMC9409192; doi:10.3390/ijms23169218)
Supplement: Supplementary file 1 [file ijms-23-09218-s001.zip › ijms-1859514-supplementary.pdf]

Supplement

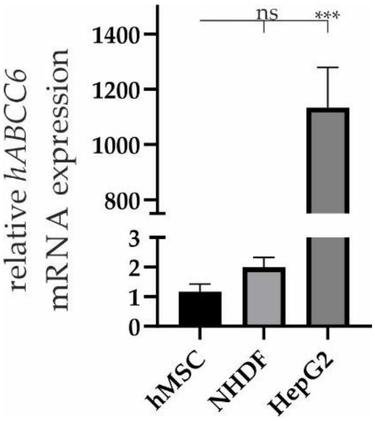

Figure S1: mRNA expressional comparison of ABCC6. ABCC6 mRNA expression in hMSCs (n = 2), normal human dermal fibroblasts (n = 2) and HepG2 (n = 1) was measured via RT-qPCR. Cells were cultured for 72 h in their specific growth medium. Data are shown as mean ± SEM. Mann-Whitney U test significance levels: not significant (ns), $p < 0.001$ (***).

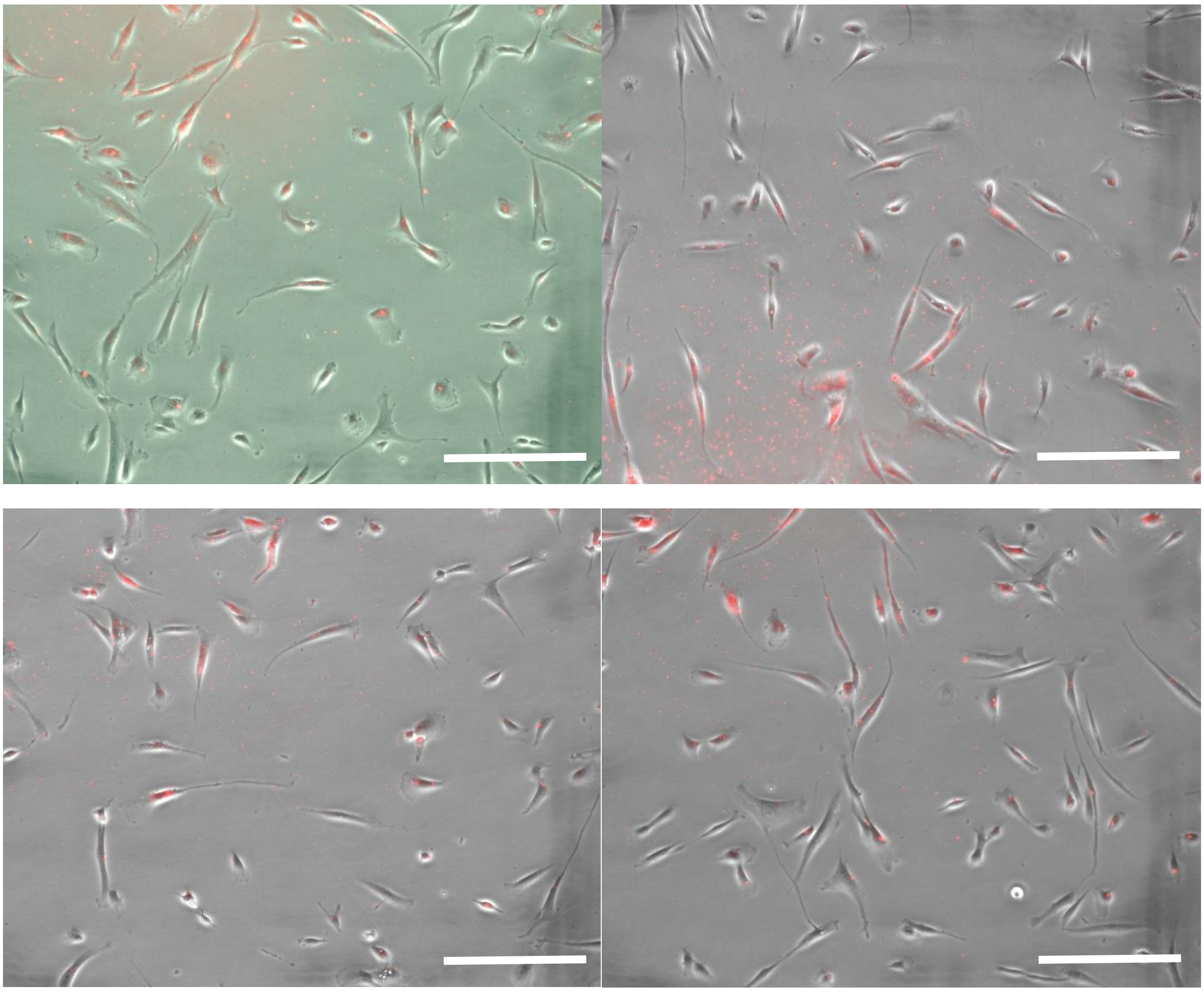

Figure S2: Representative pictures taken from transfected hMSC-68m. Fluorescence microscopy pictures were taken 24 h after transfection with RNP-complex. Scale bar: 200 µm.

Table S1: Cell counting and evaluation of ATTO 550 positive hMSC-68m from Fig. S2.

Picture	1	2	3	4
Cell count	59	55	47	56
ATTO 550 positive cells	54	52	45	52
Percentage	91.53 %	94.54 %	95.75 %	92.86 %

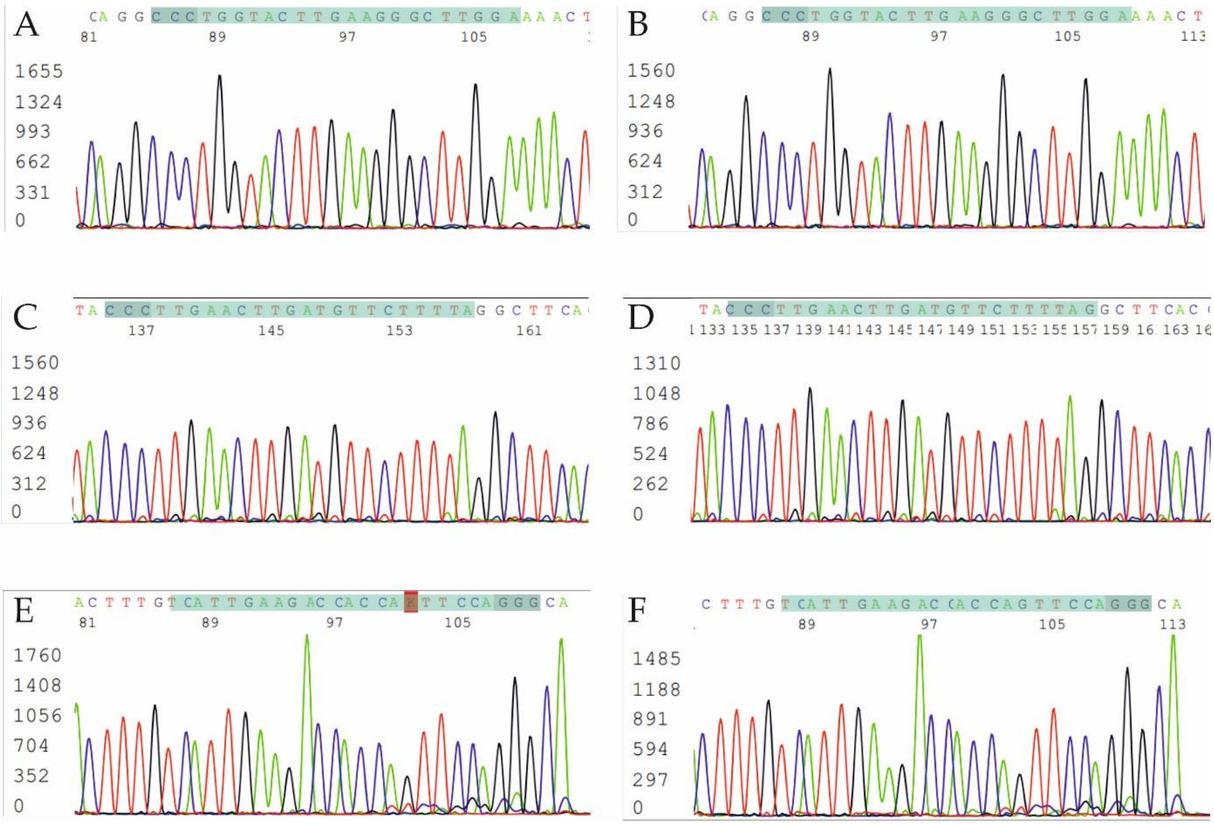

Figure S3: **Sanger sequencing of possible off-targets**. Region of potential cRNA binding is marked in green. (A+B) *GRIN1* lower strand sequencing, (C+D) *HGN5* upper strand sequencing and (E+F) *PIEZO2* upper strand sequencing for (A, C, E) *ABCC6*-deficient hMSCs and (B+D+F) wildtype hMSCs.

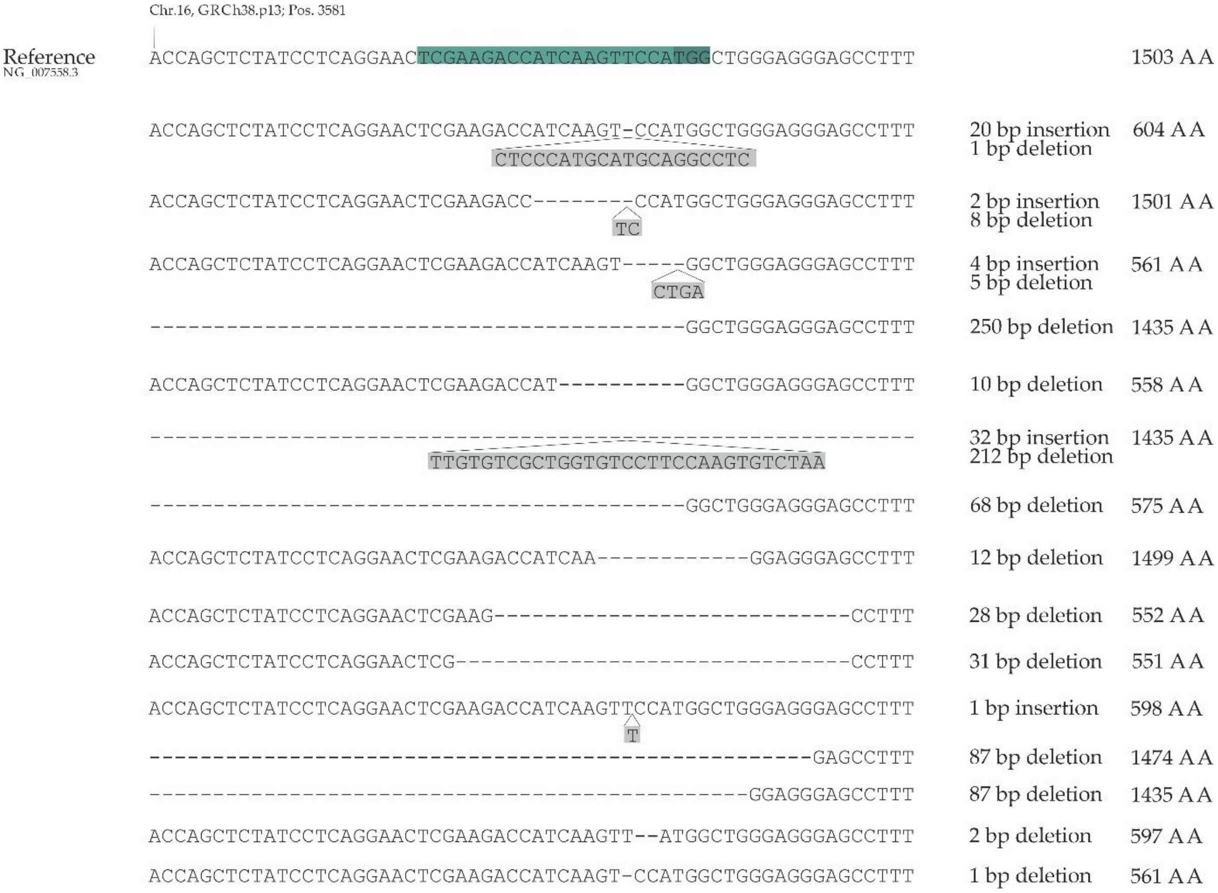

Figure S4: Variants generated by CRISPR-Cas9 knockout in ABCC6 exon 12. Sanger sequencing near gRNA sequence (green) and PAM (dark green) in hMSCs after transfection with ABCC6-specific RNP complex was performed for evaluation of mutation variants. The entry on the top presents reference sequence (NG_007558.3). Deletions are partially represented. The number of inserted or deleted base pairs (bp) and the resulting protein length is given on the right.

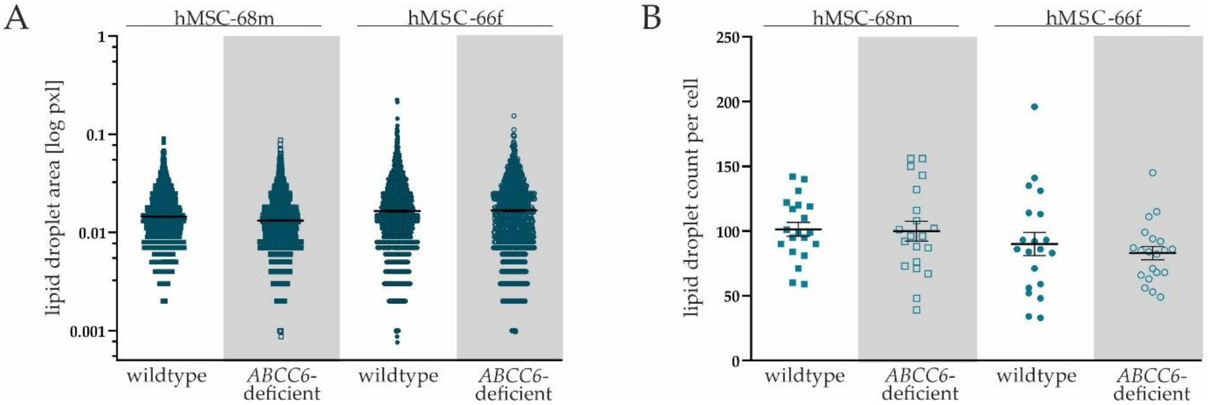

Figure S5: Lipid droplet characterization of adipocytes. Lipid droplet area and count were quantified on fluorescence microscope pictures from Bodipy staining after 19 days of differentiation for hMSC-68m and hMSC-66f separately using ImageJ. (A) Area per lipid droplet in pixels (log pxl). (B) Lipid droplet count per cell.
